# Recurrent umbilical varix rupture with hemoperitoneum: a case report and review of literature

**DOI:** 10.1186/s12876-022-02167-3

**Published:** 2022-04-01

**Authors:** W. S. Yu, M. H. Chang, H. L. Lee, Y. T. Lee, M. C. Tsai, C. C. Wang

**Affiliations:** 1 Division of Gastroenterology and Hepatology, Department of Internal Medicine, Chung Shan Medical University Hospital, Taichung, Taiwan; 2 Department of Surgery, Chung Shan Medical University Hospital, Taichung, Taiwan; 3 Infection Control, Chung Shan Medical University Hospital, Taichung, Taiwan; 4 School of Medicine, Chung Shan Medical University, Taichung, Taiwan

**Keywords:** Umbilical varix rupture, Liver cirrhosis, Hemoperitoneum, Exploratory laparotomy, Hepatocellular carcinoma

## Abstract

**Background:**

Non-traumatic hemoperitoneum was a rare event with the risk of sudden death. Spontaneous rupture of hepatocellular carcinoma is the most intuitive diagnosis when hemoperitoneum occurs in cirrhotic patients who are not regularly followed up. However, other etiologies of hemoperitoneum, such as intra-abdominal varix rupture, should be kept in mind.

**Case presentation:**

A 44-year-old man with alcoholic liver cirrhosis, Child–Pugh B was sent to our emergency department (ED) because of recurrent abdominal pain and hypovolemic shock. He had similar symptoms one month ago and was diagnosed as hepatocellular carcinoma (HCC) rupture with hemoperitoneum, therefore he underwent trans-arterial embolization (TAE). However, the follow-up magnetic resonance imaging (MRI) showed less possibility of hepatocellular carcinoma. Contrast enhanced abdominal computed tomography (CT) showed possible umbilical vein contrast agent extravasation. Exploratory laparotomy confirmed the diagnosis of rupture umbilical varix with hemoperitoneum.

**Conclusion:**

Although umbilical varix rupture is a rare cause of hemoperitoneum, it should be kept in mind in cirrhotic patients with unexplained hemoperitoneum.

**Supplementary Information:**

The online version contains supplementary material available at 10.1186/s12876-022-02167-3.

## Background

Hepatocellular carcinoma (HCC) is a late complication of chronic hepatitis B, chronic hepatitis C and alcoholic liver cirrhosis. The incidence of HCC in Taiwan is approximately 30/100,000 [1–3], which makes us remarkably familiar with the complication of HCC. In Asia, approximately 10% of patients with a diagnosis of HCC die from tumor rupture each year, and this percentage was much higher than the situation in Europe [4]. Meanwhile, spontaneous varix rupture is a lethal complication of cirrhotic patients [5–9]with portal hypertension. We presented a rare case of recurrent umbilical varix rupture with hemoperitoneum, which was initially misdiagnosed as a rupture of HCC.

## Case presentation

A 44-year-old man with a history of alcoholism and hypertension without regular medication control, presented with a sudden abdominal pain, dizziness, and loss of consciousness twice in 2 h. This patient was brought to our ED. At ED, the temperature was 36.5 °C, the blood pressure 84/42 mm Hg, the pulse 92 beats per minute, the respiratory rate 32 breaths per minute, and the oxygen saturation 95% while he was breathing ambient air. The level of consciousness was E4V4M6 but lethargy. Physical examination revealed abdominal muscle guarding with whole area rebounding pain. The lab data at ED were white blood cell (WBC) 6360/ul; hemoglobulin (Hb) 5.9 gm/dl; prothrombin time (PT) 11.9 s; aspartate aminotransferase (AST) 77 IU/l; alanine aminotransferase (ALT) 28 IU/l; total bilirubin (T/B) 1.0 mg/dl; and albumin (ALB) 2.8 gm/dl. Contrast enhanced abdominal CT presented hemoperitoneum without definite active contrast extravasation, irregular liver contour and one atypical hepatic lesion was noticed at S4 segment (Additional file 1: Figure S1). No contrast extravasation noted during emergent TAE. After emergent blood transfusion, adequate resuscitation, his condition became stable (temperature:36.7/puls:67/respiratory rate:16/blood pressure 173/108 mm Hg). Abdominal MRI revealed small liver nodules without typical hepatocellular carcinoma enhancing pattern at S2 and S4 segment of liver. The following angiography did not find the tumor and he was discharged.

**Fig. 1:**
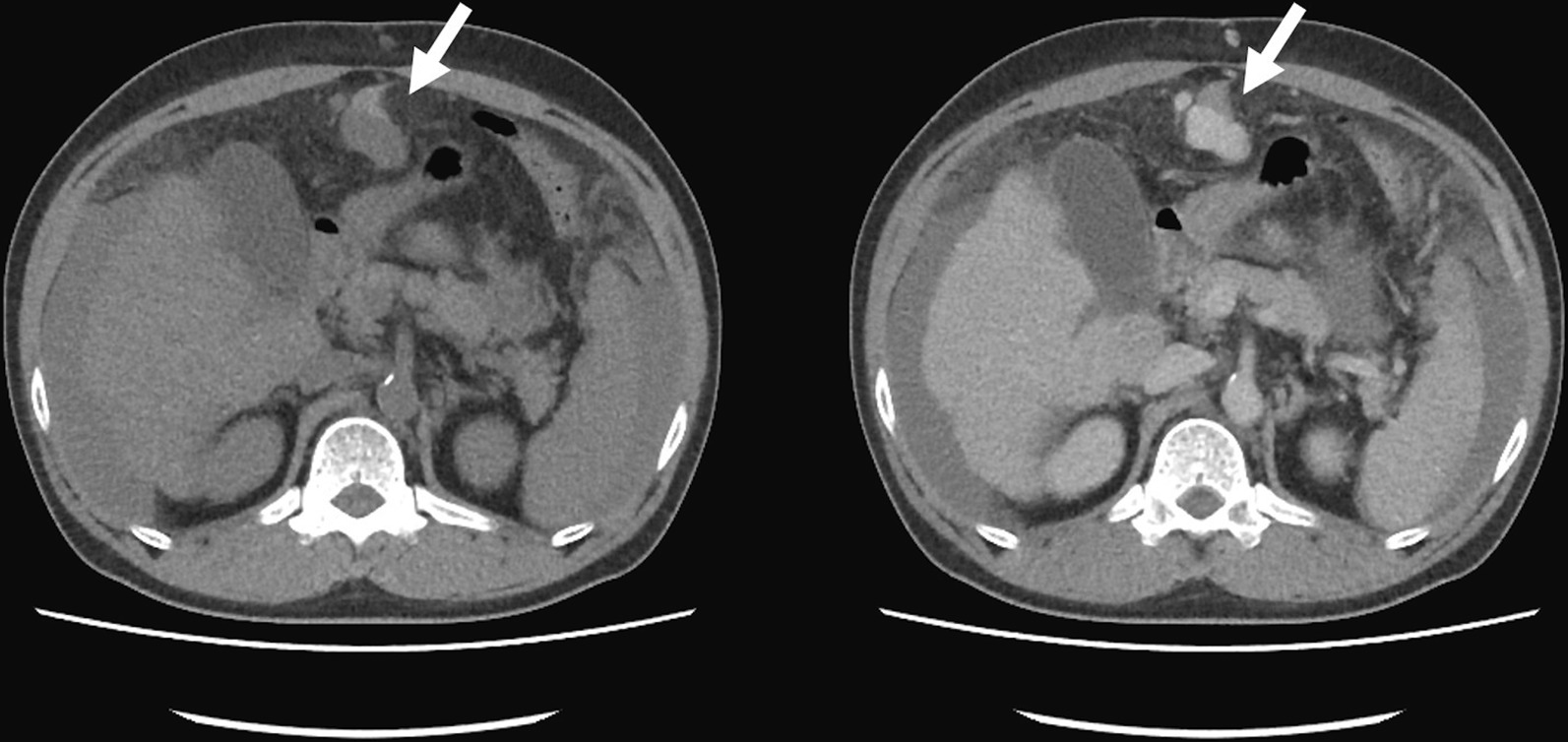
Abdominal CT demonstrated a dilated tubular structure originated from falciform ligament with contrast enhancement. In non-contrast phase, there was an adjacent high-density content beside umbilical area

**Fig. 2:**
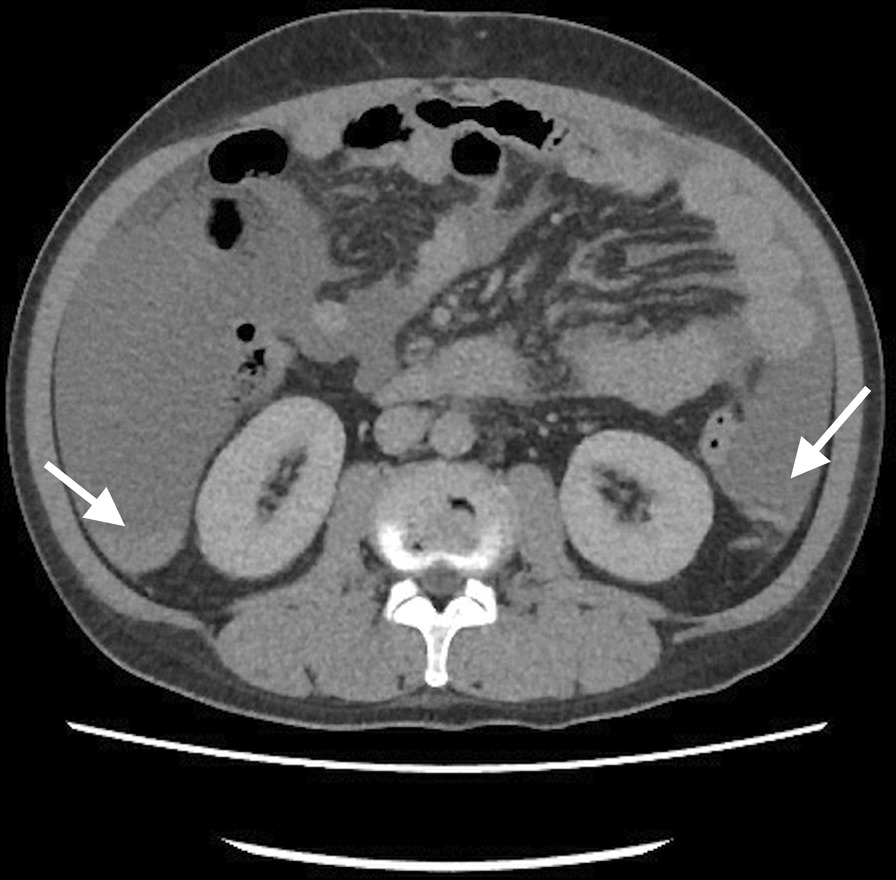
Massive high-density contents were observed inside peritoneum

**Fig. 3:**
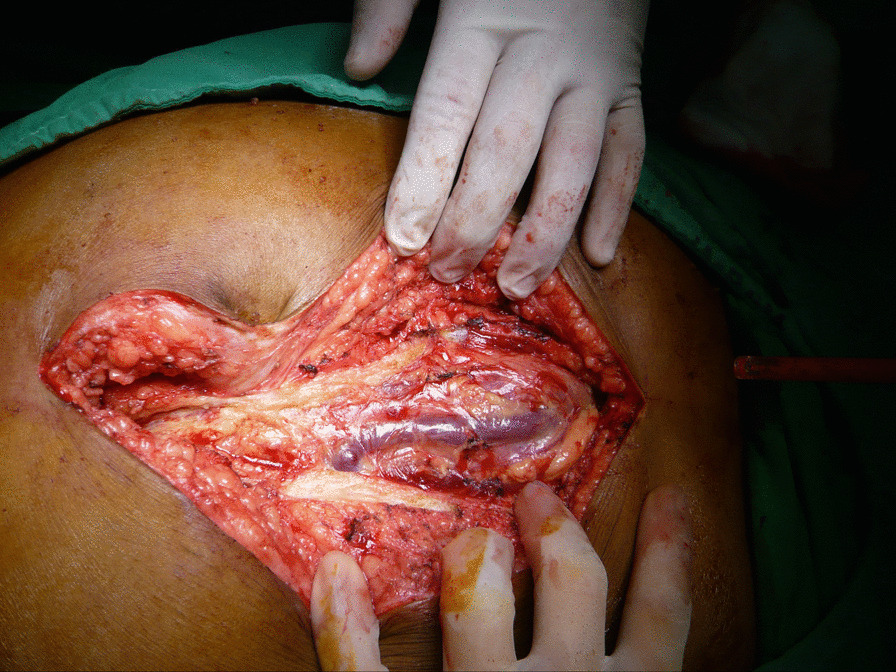
Exploratory laparotomy showed tortuous and engorged umbilical varix rupture with hemoperitoneum

Sixty-six days after last discharge, the patient was brought to our ED again due to aggravated abdominal pain and malaise for 2 days. Vital sign showed as follow: the temperature was 36.9 °C, the blood pressure 77/44 mm Hg, the pulse 95 beats per minute, the respiratory rate 19 breaths per minute, and the oxygen saturation 97% while he was breathing ambient air. The level of consciousness was E4V4M6 but obtundation. The blood test at ED showed WBC 9140/ul; Hb of 6.9 gm/dl; PT 10.9 s; AST 27 IU/l; ALT 20 IU/l; T/B 1.1 mg/dl; and ALB 2.3 gm/dl. Sonography showed massive ascites, and bloody contents were observed during abdominal paracentesis. Abdominal CT demonstrated a dilated tubular structure originated from falciform ligament with contrast enhancement. In non-contrast phase, there was an adjacent high-density content beside umbilical area (Fig. 1) and massive high-density contents were observed inside peritoneum (Fig. 2). Those high-density contents were suspected to be blood clots. Exploratory laparotomy showed tortuous and engorged umbilical varix rupture with hemoperitoneum (Fig. 3). Active oozing was noticed, and the ligation was successfully completed without complication (Additional file 2: Video S1). One week after the operation, the patient was discharged in a stable condition. No more spontaneous hemoperitoneum or hypovolemic shock was detected. The patient felt satisfied and received regular follow-up at our out-patient clinics due to the high recurrence rate of varicose vein rupture.

## Discussion and conclusions

Non-traumatic hemoperitoneum in patients with cirrhosis is fatal and emergent. When spontaneous hemoperitoneum occurs, we should evaluate the possibilities of hepatocellular carcinoma, ovarian carcinoma, hemorrhagic pancreatitis, and rupture of intra-abdominal varices [1, 3]. If this condition happens in patients with liver cirrhosis, the possibility of HCC rupture or intra-abdominal varix rupture should be considered first. In the west, the rate of HCC rupture was less than 3% [2, 4, 9], but the rates of HCC rupture was much more higher in Asian countries (10% from Japan, 12.4% from Thailand, 12.7% from southern Africa, 14.5% from Hong Kong, 26% from Taiwan) [2, 5–8, 10, 11]. Meanwhile, the true incidence of intra-abdominal varix rupture is still unknown. A past article review showed that in 18% of spontaneous hemoperitoneum, rupture of peritoneal varix were detected on abdominal CT scan, but this reference was limited to number of cases and the reference was outdated [12]. Patients with liver cirrhosis are more likely to develop HCC rupture than rupture of intra-abdominal varix.

There is no meta-analysis or systemic review for the investigation of intra-abdominal varix rupture [13]. However, some articles suggest that abdominal contrast CT scan can clarify whether this is a tumor or varices bleeding [14, 15]. Paracentesis with hematocrit ratio of more than 5% leads to the diagnosis of definite hemoperitoneum rather than intra-gastrointestinal bleeding and it is an indication for laparotomy [3]. Angiography was hard to identify the true bleeding vessel and therefore postpone the operation, which lead to increase mortality rate of patient [16]. However, an angiography with TAE plays an important role in conservative treatment [17]. Some articles presented less mortality rate (78% to 57%) if emergency laparotomy is performed with varix ligation [16].

Based on the surgical review, in 20% cases of varies rupture related hemoperitoneum caused by recanalized umbilical vein [16]. To the embryology, the umbilical vein is quickly completely obliterated and turns ligamentum teres hepatis [18]. During the progression of liver cirrhosis with portal hypertension, the pressure forced the blood pressing against the ligament to recanalized umbilical vein, which is nearby hypogastric and iliac vein [19].

Back to our case, there was no case report presented both liver nodular lesion and co-existed varix. Rupture of HCC with self-limited bleeding listed to the most possible diagnosis in this patient without records of previous esophageal or gastric varices bleeding. Nevertheless, we ought to consider the possibility of intra-abdominal varix rupture when the image of MRI revealed HCC less likely. This case informed us that we should keep in mind of the diagnosis of umbilical varix rupture in patient of spontaneous hemoperitoneum.

## Supplementary Information

**Additional file 1**. The result of contrast enhanced abdominal CT showed one atypical hepatic lesion at S4 segment.

**Additional file 2**. This is a supplementary video demonstrating tortuous and engorged umbilical varix rupture with active oozing was ligated successfully through exploratory laparotomy.

## Data Availability

Not applicable because of this is a case report.

## Abbreviations

ED: Emergency department
HCC: Hepatocellular carcinoma
TAE: Trans-arterial embolization
MRI: Magnetic resonance imaging
CT: Computed tomography
WBC: White blood cells
Hb: Hemoglobulin
PT: Prothrombin time
AST: Aspartate aminotransferase
ALT: Alanine aminotransferase
T/B: Total bilirubin
ALB: Albumin
