# Supplementary figures and images for: Recurrent umbilical varix rupture with hemoperitoneum: a case report and review of literature
Source: BMC Gastroenterol. 2022 Apr 1;22:160. doi: 10.1186/s12876-022-02167-3 (PMC8973573; doi:10.1186/s12876-022-02167-3)

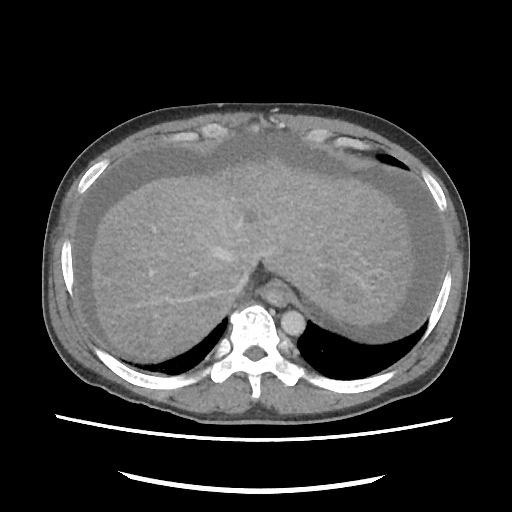

Supplement: Supplementary file 1 — Additional file 1. The result of contrast enhanced abdominal CT showed one atypical hepatic lesion at S4 segment. [file 12876_2022_2167_MOESM1_ESM.jpg]
